# Mortality prediction in hemodialysis patients using heart rate variability and skin sympathetic nerve activity

**DOI:** 10.1080/0886022X.2025.2596442

**Published:** 2026-01-20

**Authors:** Xinyi Fu, Yaoyu Huang, Yujun Qian, Shuang Su, Yike Zhang, Zhenye Chen, Hongwu Chen, Ming Zeng, Jing Wang, Huijuan Mao

**Affiliations:** ^a^ Department of Nephrology, The First Affiliated Hospital with Nanjing Medical University, Jiangsu Province Hospital, Nanjing, China; ^b^ Department of Nephrology, Nanjing Pukou People’s Hospital, Nanjing, China; ^c^ Department of Cardiology, The First Affiliated Hospital with Nanjing Medical University, Jiangsu Province Hospital, Nanjing, China

**Keywords:** Risk stratification, autonomic dysfunction, dialysis prognosis, nomogram, machine learning, LASSO regression

## Abstract

Patients undergoing maintenance hemodialysis (HD) face a substantially elevated risk of all-cause mortality, yet robust tools for individualized risk stratification remain limited. This multicenter study developed a predictive model integrating dynamic autonomic nervous system (ANS) markers – heart rate variability (HRV) and skin sympathetic nerve activity (SKNA) – with clinical factors to assess mortality risk. We enrolled 198 HD patients from two Chinese centers between 2021 and 2023, recording HRV/SKNA parameters at baseline, 30 min, and 240 min into dialysis. Over a median follow-up of 34 months, the all-cause mortality rate was 17.7%. Ninety-one baseline features were included in the LASSO-regression model. The final multivariable logistic regression model incorporated six variables (diabetes mellitus, DBP_2h_, RMSSD_240_, ΔNnmean_30_, ΔApEn_30_ and ΔaSKNA_240_) into the nomogram. The AUC of the nomogram for predicting one-year, two-year, and three-year survival rates was 0.764, 0.749, and 0.805, respectively. The Kaplan–Meier curves for overall survival stratified by nomogram model showed a significant difference between high- and low- risk groups. Internal validation *via* bootstrap resampling confirmed model robustness, with optimism-corrected AUCs of 0.758, 0.736, and 0.788 for one-, two-, and three-year mortality, respectively. The model demonstrated superior predictive accuracy for cardiovascular mortality (C-index = 0.881) and consistent performance across age and sex subgroups. The proposed model has the potential to predict all-cause mortality in HD patients and may enable earlier intervention and personalized management.

## CLINICAL PERSPECTIVES

- Patients undergoing maintenance HD face high mortality risks, yet existing risk stratification tools lack integration of dynamic ANS markers. This study developed a predictive model integrating intradialytic HRV/SKNA parameters with clinical factors to improve risk assessment.
- The LASSO-based nomogram identified six predictors (diabetes mellitus, DBP_2h_, RMSSD_240_, ΔNnmean_30_, ΔApEn_30_ and ΔaSKNA_240_) and achieved AUCs of 0.764, 0.749, and 0.805 for one-year, two-year and three-year mortality prediction respectively, with significant stratification of high- and low-risk groups (*p* < .0001).
- This model facilitates early identification of high-risk MHD patients, offering a practical tool to guide personalized interventions and potentially reduce mortality through targeted management of ANS dysfunction. Moreover, the findings highlight the critical role of dialysis-induced autonomic instability in long-term outcomes.

## Introduction

Chronic kidney disease (CKD) imposes a significant socioeconomic burden and is associated with increased mortality. Epidemiological data show that approximately 14.0% of adults in the United States and 8.9% of adults in mainland China suffer from CKD [1,2]. Patients receiving maintenance hemodialysis (HD) continue to face persistently high mortality risk. According to the United States Renal Data System (USRDS), the adjusted all-cause mortality rate in 2022 remained as high as 145.6 per 1,000 person-years, indicating an annual mortality rate approaching 15% in the dialysis population [3]. In response to this substantial burden, the 2024 Kidney Disease: Improving Global Outcomes (KDIGO) Clinical Practice Guideline for the Early Evaluation and Management of Chronic Kidney Disease has emphasized a paradigm shift toward proactive risk stratification. It specifically calls for the integration of novel physiological markers to enable personalized care and improve clinical outcomes [4]. Previous studies have indicated that patients with CKD often exhibit autonomic nervous system (ANS) alterations characterized by upregulated sympathetic activity and downregulated parasympathetic activity, which is associated with various adverse outcomes in dialysis patients [5,6].

Heart rate variability (HRV) is a diagnostic measure reflecting ANS function. Prior research and meta-analyses have demonstrated that reduced HRV parameters are associated with increased risk of all-cause mortality and cardiovascular events [7]. As a robust predictor of cardiovascular disease onset and prognosis, HRV has been utilized to forecast long-term mortality in dialysis patients and the occurrence of intradialytic hypotension [7,8]. Skin sympathetic nerve activity (SKNA), a novel method proposed in recent years for assessing cardiac sympathetic activity, has been experimentally validated to exhibit linear correlation with direct recordings of stellate ganglion neural activity in animal studies [9], though its clinical application in dialysis populations remains limited [10].

Moreover, previous studies have not demonstrated the correlation between HRV/SKNA dynamic changes and all-cause mortality during maintenance hemodialysis (HD). The study aimed to identify dynamic changes in HRV and SKNA in HD patients and to develop a nomogram model for predicting all-cause mortality in HD patients.

## Results

### Baseline characteristics of participants

**Figure 1.**
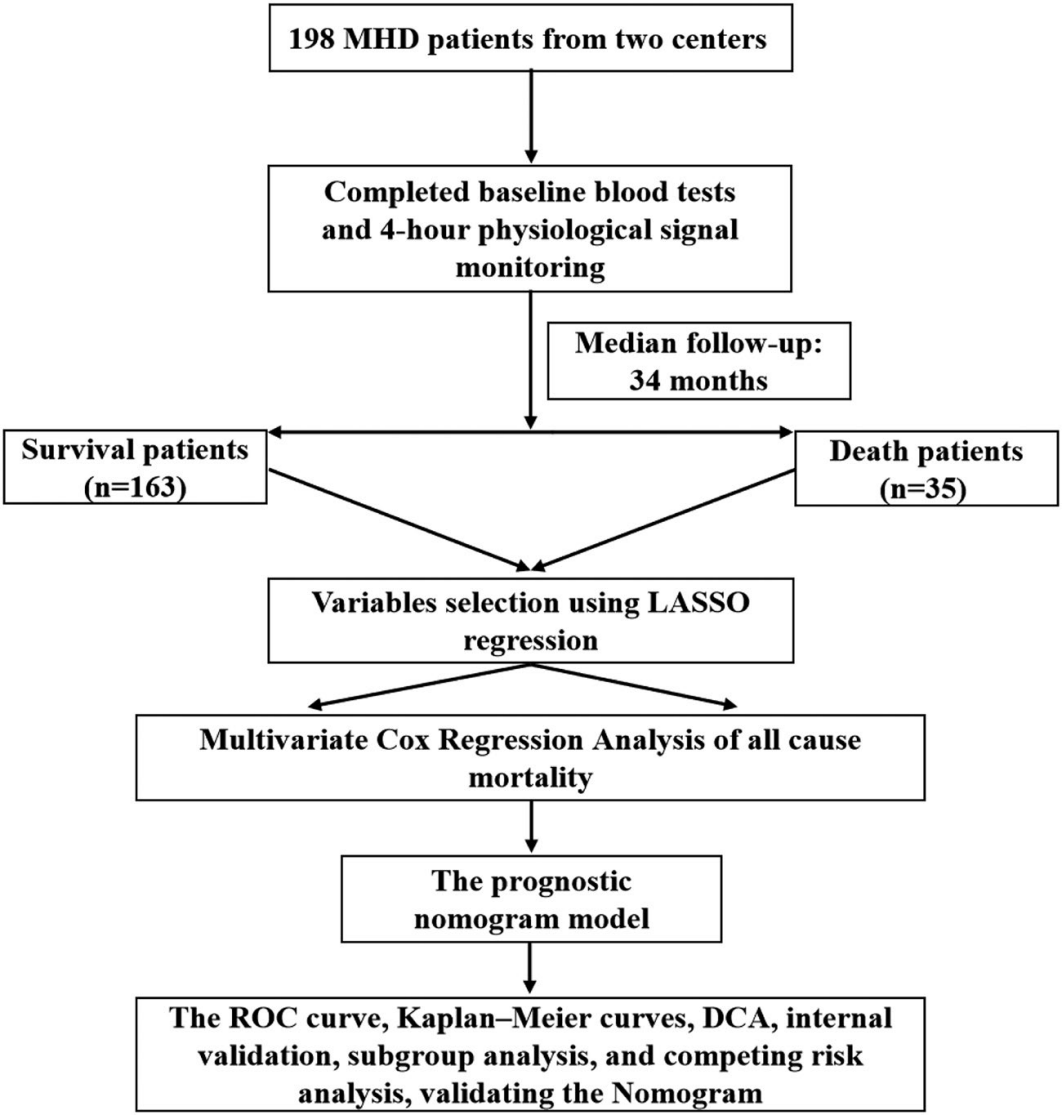
Flowchart of hemodialysis patient enrollment for mortality prediction using HRV and SKNA parameters. The workflow illustrates cohort formation, predictor selection, nomogram construction, and multi-faceted model validation.

After median 34 months follow-up, a total of 198 MHD patients from two separate centers (100 from Center 1 and 98 from Center 2) were divided into two subgroups: 35 non-survivors and 163 survivors. The flowchart of this study was demonstrated in Figure 1. The demographic and baseline characteristics were detailed in Table 1. There were 94 women and 104 men enrolled in our study, the average age was 59.97 ± 12.94 years old, and median dialysis vintage was 9.00 (3.00, 59.00) months. Compared with survival group, the patients with all-cause mortality had older age (65.29 ± 12.73 vs. 58.83 ± 12.73 years, *p* = .007), lower serum albumin levels (38.00 [33.40, 41.05] vs. 39.40 [37.70, 42.15] g/L, *p* = .015), higher prevalence of diabetes mellitus (16/35 vs. 37/163, *p* = .010), lower serum phosphorus levels (1.71 ± 0.51 vs. 1.92 ± 0.50 mmol/L, *p* = .028) and higher C-reaction protein (5.48 [2.45, 9.13] vs. 3.00 [1.35, 6.34] mg/L, *p* = .022). The baseline demographics, clinical characteristics, and laboratory results in survival and all-cause mortality subgroup of HD patients are summarized in Table 1.

**Table 1.** Demographic and baseline characteristics in survivors and non-survivors of CKD5 patients.

Clinical characteristics	Overall (*n* = 198)	Survivors (*n* = 163)	Non-survivors (*n* = 35)	*p* Value
Center, *n* (%)				
1	100	83	17	
2	98	80	18	
Demographics				
Female/male (%)	94 (47.47)	76 (46.63)	18 (51.43)	.742
Age (years)	59.97 ± 12.94	58.83 ± 12.73	65.29 ± 12.73	.007
BMI (kg/m^2^)	22.8 ± 3.50	22.9 ± 3.57	22.0 ± 3.10	.136
SBP (mmHg)	145.02 ± 21.65	144.57 ± 23.01	147.03 ± 13.74	.293
DBP (mmHg)	78.50 ± 11.98	79.07 ± 12.12	75.89 ± 10.95	.149
IDH (%)	89 (44.95)	71 (43.56)	18 (51.43)	.508
Dialysis vintage (months)	9.00 (3.00, 59.00)	9.00 (3.00, 60.50)	8.00 (3.00, 49.00)	.921
IDWG (%)	2.50 (1.60, 3.00)	2.50 (1.65, 3.00)	2.20 (1.55, 3.10)	.378
IDWG (kg)	2.50 (1.72, 3.00)	2.50 (1.80, 3.00)	2.20 (1.60, 2.60)	.148
Ultrafiltration (L)	2.50 (1.72, 3.00)	2.50 (1.80, 3.00)	2.20 (1.60, 2.60)	.155
Comorbidities, *n* (%)				
Diabetic mellitus	53 (26.77)	37 (22.70)	16 (45.71)	.010
Hypertension	159 (80.30)	128 (78.53)	31 (88.57)	.262
Polycystic kideny disease	7 (3.53)	7 (4.29)	0 (0.00)	.357
Medication history, *n* (%)				
ACEI/ARB	57 (28.79)	47 (28.83)	10 (28.57)	1.000
β-receptor blockers	73 (36.87)	60 (36.81)	13 (37.14)	.958
Phosphate binders	81 (40.91)	73 (43.56)	8 (28.57)	.027
Activated vitamin D or analogs	81 (40.91)	70 (42.94)	11 (31.43)	.285
Laboratory values				
Hemoglobin (g/L)	103.10 ± 17.11	103.56 ± 16.86	100.91 ± 18.32	.407
Total cholesterol (mmol/L)	4.02 (3.40, 4.76)	4.04 (3.42, 4.71)	3.70 (3.18, 4.83)	.157
Triglyceride (mmol/L)	1.79 (1.23, 2.57)	1.84 (1.28, 2.59)	1.69 (1.16, 2.50)	.505
Albumin (g/L)	39.20 (36.82, 41.90)	39.40 (37.70, 42.15)	38.00 (33.40, 41.05)	.015
Calcium (mmol/L)	2.19 ± 0.23	2.20 ± 0.23	2.18 ± 0.23	.621
Phosphorus (mmol/L)	1.88 ± 0.51	1.92 ± 0.50	1.71 ± 0.51	.028
ALP (U/L)	93.00 (71.62, 126.15)	93.00 (68.90, 123.10)	98.20 (83.40, 136.80)	.183
iPTH (pg/mL)	243.65 (109.00, 471.43)	256.00 (124.00, 482.30)	157.90 (81.25, 332.70)	.095
CRP	3.21 (1.41, 7.38)	3.00 (1.35, 6.34)	5.48 (2.45, 9.13)	.022

*Note:* Data are presented as mean ± *SD*, medians with interquartile ranges (IQRs), numbers and percentages, as appropriate.

Abbreviations: CKD: chronic kidney disease; BMI: body mass index; SBP: systolic blood pressure; DBP: diastolic blood pressure; IDH: intradialytic hypotension; Polycy: polycystic kidney disease; IDWG: interdialytic weight gain; ACEI/ARB: angiotensin-converting enzyme inhibitors /angiotensin receptor blocker; ALP: alkaline phosphatase; iPTH: intact parathyroid hormone; CRP: C-reaction protein.

### Baseline HRV parameters in HD patients

**Figure 2.**
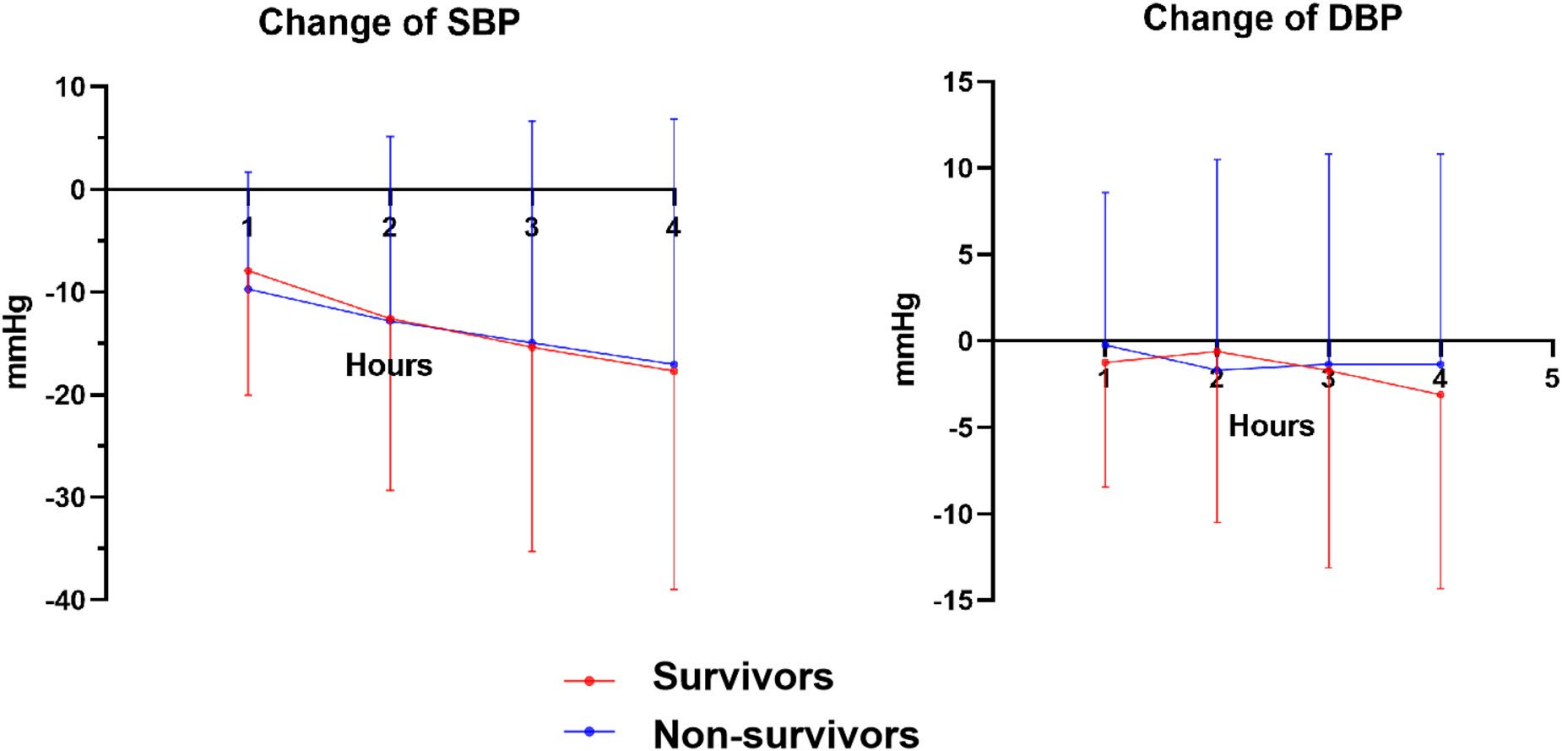
Changes in systolic and diastolic blood pressure during hemodialysis. Blood pressure measurements were recorded hourly after dialysis initiation. Data are presented as mean ± standard deviation, as appropriate.

Representative HRV indices and average skin sympathetic nerve activity (aSKNA) values recorded at three time points, along with their dynamic changes between different time points, are presented in Table 2. All abbreviations are consistent with those defined in the Methods section. Compared with non-survivors, there were significantly higher SDNN (21.98 [18.57, 30.36] vs. 28.26 [20.71, 38.38], *p* = .041), TTLPWR (495.10 [345.38, 945.83] vs. 812.71 [460.15, 1,455.05], *p* = .012], VLF (151.09 [97.87, 312.99] vs. 344.16 [171.30, 574.61], *p* < .001), LF (61.79 [29.84, 136.09] vs. 146.18 [67.82, 307.16], *p* < .001), and LF/HF (0.57 [0.26, 0.91] vs. 1.07 [0.57, 1.92], *p* < .001) in survivors. SKNA_240_ (20.49 [15.82, 31.21] vs. 27.73 [19.24, 37.44], *p* = .021), TTLPWR_240_ (352.52 [233.64, 852.02] vs. 889.39 [444.52, 1,577.78], *p* = .001), VLF_240_ (160.57 [90.64, 300.03] vs. 404.20 [199.48, 798.67], *p* < .001), LF_240_ (47.95 [28.32, 160.55] vs. 159.58 [74.42, 351.35], *p* < .001), LF/HF_240_ (0.54 [0.29, 0.96] vs. 1.38 [0.68, 3.15], *p* < .001) and DC_240_ (2.35 [1.59, 3.69] vs. 3.86 [2.51, 5.99], *p* = .002)were also higher in survival subgroup. Regarding dynamic changes of ANS, non-survivors had higher ΔMHR_30_ (0.01 [−0.01, 0.05] vs. −0.01 [−0.05, 0.01], *p* < .001), ΔApEn_240_ (0.10 [−0.10, 0.25] vs. 0.00 [−0.11, 0.10], *p* = .034) and ΔaSKNA_240_ (0.02 [−0.09, 0.14] vs. −0.04 [−0.14, 0.05], *p* = .028), but lower ΔHF_30_ (−0.35 [−0.68, 0.10] vs. −0.06 [−0.58, 0.54], *p* = .038) compared to survival subgroup. Moreover, change of SBP and DBP were shown in Figure 2. Complete HRV data are summarized in Supplementary Tables 1–5.

**Table 2.** Representative HRV indexes in survivors and non-survivors of CKD5 patients.

Heart rate variability	Overall (*n* = 200)	Survivors (*n* = 164)	Non-survivors (*n* = 36)	*p* Value
△MHR_30_ (bpm)	−0.01 (−0.04, 0.02)	−0.01 (−0.05, 0.01)	0.01 (−0.01, 0.05)	<.001
SDNN (ms)	26.41 (20.17, 36.68)	28.26 (20.71, 38.38)	21.98 (18.57, 30.36)	.041
SDNN_240_ (ms)	26.07 (18.62, 36.70)	27.73 (19.24, 37.44)	20.49 (15.82, 31.21)	.021
TTLPWR	726.72 (420.81, 1,388.05)	812.71 (460.15, 1,455.05)	495.10 (345.38, 945.83)	.012
TTLPWR_240_	744.71 (356.38, 1,399.98)	889.39 (444.52, 1,577.78)	352.52 (233.64, 852.02)	.001
VLF	321.73 (138.97, 558.53)	344.16 (171.30, 574.61)	151.09 (97.87, 312.99)	<.001
VLF_240_	358.61 (161.61, 731.43)	404.20 (199.48, 798.67)	160.57 (90.64, 300.03)	<.001
LF	127.02 (59.36, 281.92)	146.18 (67.82, 307.16)	61.79 (29.84, 136.09)	<.001
LF_240_	141.55 (58.46, 311.85)	159.58 (74.42, 351.35)	47.95 (28.32, 160.55)	<.001
△HF_30_	−0.10 (−0.61, 0.35)	−0.06 (−0.58, 0.54)	−0.35 (−0.68, 0.10)	.044
LF/HF	0.92 (0.48, 1.77)	1.07 (0.57, 1.92)	0.57 (0.26, 0.91)	<.001
LF/HF_240_	1.13 (0.58, 2.84)	1.38 (0.68, 3.15)	0.54 (0.29, 0.96)	<.001
DC_240_	3.49 (2.21, 5.77)	3.86 (2.51, 5.99)	2.35 (1.59, 3.69)	.002
△ApEn_240_	0.01 (−0.11, 0.12)	0.00 (−0.11, 0.10)	0.10 (−0.10, 0.25)	.034
△aSKNA_240_	−0.03 (−0.13, 0.07)	−0.04 (−0.14, 0.05)	0.02 (−0.09, 0.14)	.028

Abbreviations: HRV: heart rate variability; CKD: chronic kidney disease; MHR: mean heart rate; SDNN: standard deviation of normal-to-normal intervals; TTLPWR: total power; VLF: very low-frequency power; LF: low-frequency power; HF: high-frequency power; LF/HF: the ratio of low to high-frequency power; DC: deceleration capacity; ApEn: approximate entropy; aSKNA: average skin sympathetic nerve activity.

**Table 3.** LASSO-derived multivariate logistics regression analysis of clinical risk factors for all-cause mortality in CKD5 patients.

	Coefficients	Standard error	Wald Z	*p* Value
Diabetic mellitus	0.962	0.552	1.742	.081
Phosphate binders	－0.468	0.561	－0.835	.404
DBP_2h_	－0.044	0.023	－1.935	.053
Albumin	－0.068	0.052	－1.289	.197
Phosphorus	－0.105	0.565	－0.185	.853
VLF	－0.001	0.001	－1.427	.153
LF/HF	－0.389	0.392	－0.993	.321
△aSKNA_240_	2.627	1.411	1.862	.063
rMSSD_240_	0.035	0.016	2.129	.033
ULF_240_	－0.005	0.004	－1.076	.282
VLF_240_	0.000	0.001	0.018	.985
LF/HF_240_	－0.409	0.353	－1.157	.247
△VLF_240_	－0.482	0.331	－1.459	.144
△ApEn_240_	－0.959	1.401	－0.685	.493
△NNmean_30_	－19.100	5.170	－3.695	.000
△ApEn_30_	2.484	1.493	1.663	.096

Abbreviations: DBP: diastolic blood pressure; VLF: very low-frequency power; LF/HF: the ratio of low to high-frequency power; aSKNA: average skin sympathetic nerve activity; rMSSD: root mean square of successive differences; ULF: ultra low-frequency power; ApEn: approximate entropy; NNmean: mean of all normal-to-normal intervals during 24 h.

**Table 4.** Multivariate Cox regression analysis of clinical risk factors for all-cause mortality in CKD5 patients.

	HR (95%Cl)	*p* Value
Diabetes mellitus	2.73 (1.34, 5.58)	.006
DBP_2h_	0.97 (0.94, 0.99)	.021
△aSKNA_240_	4.31 (1.00, 18.55)	.049
rMSSD_240_	1.02 (1.00, 1.03)	.011
△NNmean_30_	0.00 (0.00, 0.26)	.010
△ApEn_30_	4.49 (1.36, 14.82)	.013

Abbreviations: DBP: diastolic blood pressure; aSKNA: average skin sympathetic nerve activity; rMSSD: root mean square of successive differences; ApEn: approximate entropy; NNmean: mean of all normal-to-normal intervals during 24 h.

### Variables selection using LASSO regression and logistic regression

**Figure 3.**
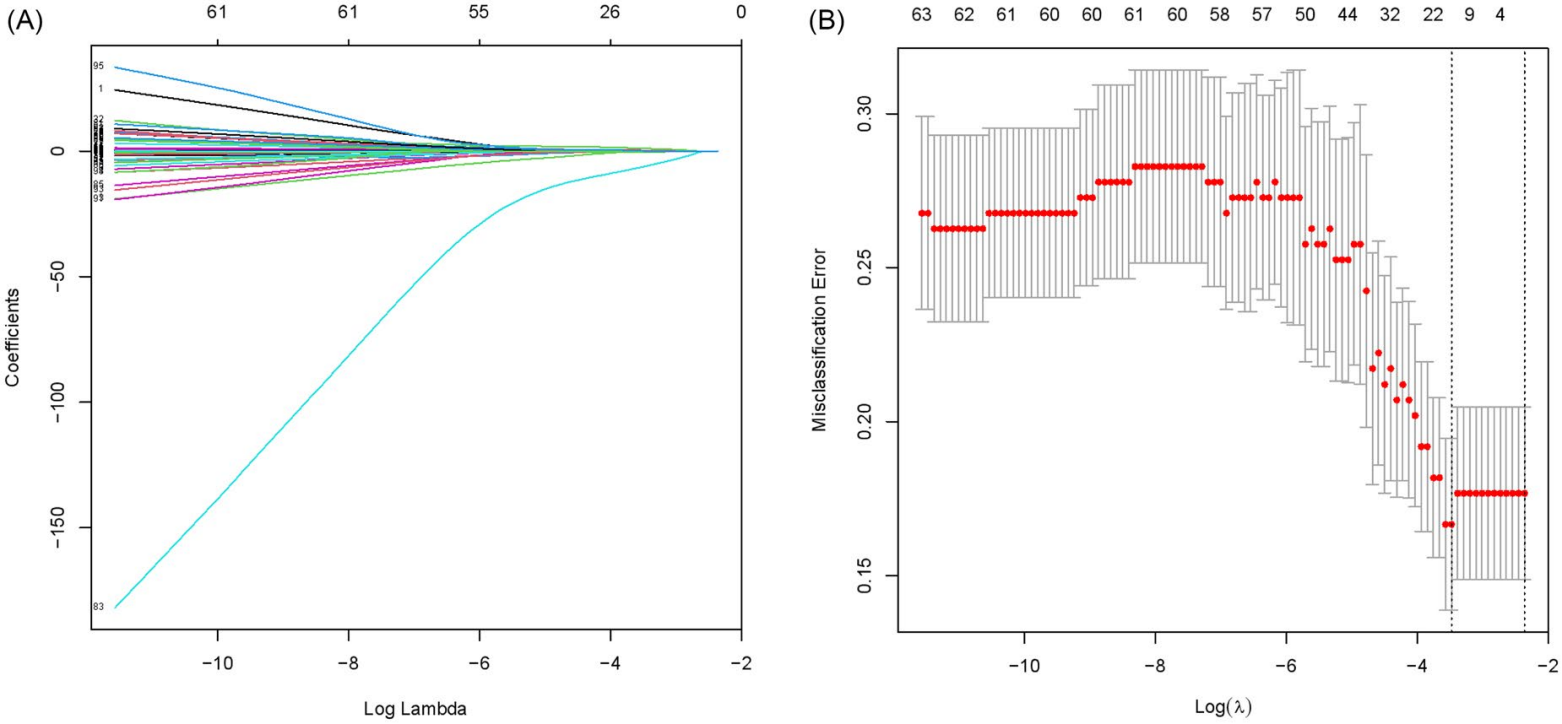
Variable selection using LASSO regression. (A) LASSO coefficient profiles of the 91 candidate variables. (B) Ten-fold cross-validation for tuning parameter (λ) selection in the LASSO model. The left vertical line indicates the λ value with minimum mean cross-validated error, and the right vertical line indicates the λ value within one standard error of the minimum.

Least absolute shrinkage and selection operator (LASSO) regression, which is known to be able to remove unimportant variables *via* the regression coefficients penalizing the size of the parameters, has been extended and broadly applied to the Cox proportional hazards model for survival analysis. Finally, a λ value of 0.2, with log (λ), −1.6 was chosen for overall survival (OS) analysis (Figure 3(A,B)). 91 variables measured were included in the LASSO regression analysis. After LASSO regression selection (Figure 3), the following 16 variables remained significant predictors of all-cause mortality, including diabetes mellitus, phosphate binders, DBP_2h_, albumin, phosphorus, ΔaSKNA_240_, VLF, LF/HF, rMSSD_240_, ULF_240_, VLF_240_, LF/HF_240_, ΔVLF_240_, ΔApEn_240_, ΔNnmean_30_, ΔApEn_30_. These 16 variables were included in the multivariate logistic regression model, and ultimately 6 variables were found to be significant predictors of all-cause mortality at a threshold of *p* < .1 (Table 3). To further validate the prognostic significance of these predictors, we performed multivariate Cox proportional hazards analysis (Table 4). All six variables retained in the logistic regression model independently predicted all-cause mortality in the Cox model. The consistency between LASSO-logistic and Cox regression results confirmed the robustness of these predictors for mortality risk stratification.

### Nomogram model establishment and predicting all-cause mortality

**Figure 4.**
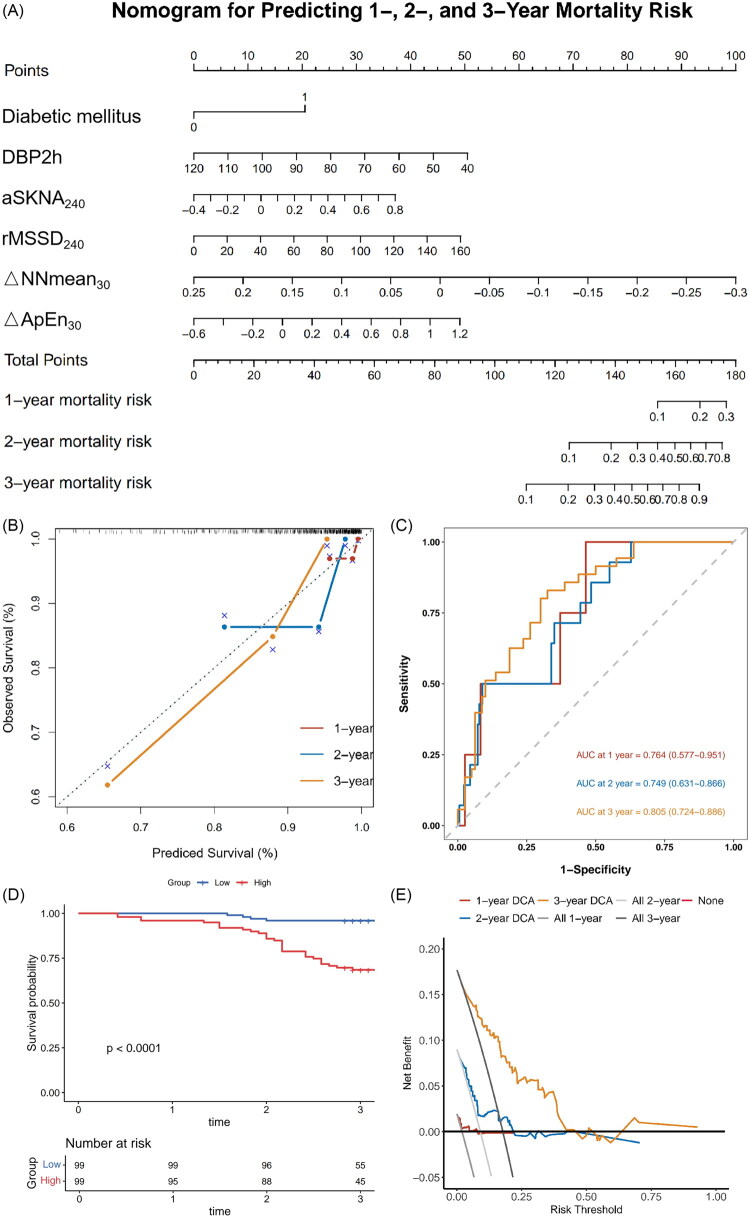
Nomogram development and validation. Nomogram for predicting one-year, two-year, and three-year mortality risk in hemodialysis patients. The nomogram incorporates six predictors: diabetes mellitus, DBP_2h_, RMSSD_240_, ΔNnmean_30_, ΔApEn_30_, and ΔaSKNA_240_. (B) Calibration curves for one-year, two-year, and three-year survival predictions. The dashed line represents the ideal fit. (C) ROC curves showing the predictive performance of the nomogram one-year, two-year, and three-year mortality. (D) Kaplan–Meier survival curves stratified by the nomogram-based risk groups (high-risk vs. low-risk). Log-rank test was used to compare survival between groups. (E) Decision curve analysis (DCA) demonstrating the net clinical benefit of the nomogram across a range of threshold probabilities.

Six independent prognostic variables for all-cause mortality were identified through multivariate logistic regression. To calculate the weight of each selected factor, a nomogram was generated in Figure 4(A), which was used to predict the one-year, two-year, and three-year mortality risk of patients in the early stages of hemodialysis. The calibration curves (Figure 4(B)) demonstrated excellent agreement between predicted survival and observed survival at all time points, validating the model’s accuracy. The prediction performance of the nomogram was further evaluated by area under the curve (AUC) analysis. The AUC for one-year mortality was 0.76 (95% CI: 0.58–0.95) with sensitivity of 100% and specificity of 53.61%, for two-year mortality was 0.75 (95% CI: 0.63–0.87) with sensitivity of 50.00% and specificity of 91.11%, and for three-year mortality was 0.80 (95% CI: 0.72–0.89) with sensitivity of 82.94% and specificity of 67.50%, respectively. Kaplan–Meier analysis (Figure 4(D)) stratified by the nomogram’s risk groups revealed significant divergence in overall survival between high- and low-risk patients (*p* < .0001), confirming the model’s ability to stratify mortality risk. Decision curve analysis (Figure 4(E)) demonstrated that the model provided net clinical benefits across a range of risk-threshold probabilities, supporting its clinical utility for guiding clinical decision-making in this population.

### Model validation and comparison

**Figure 5.**
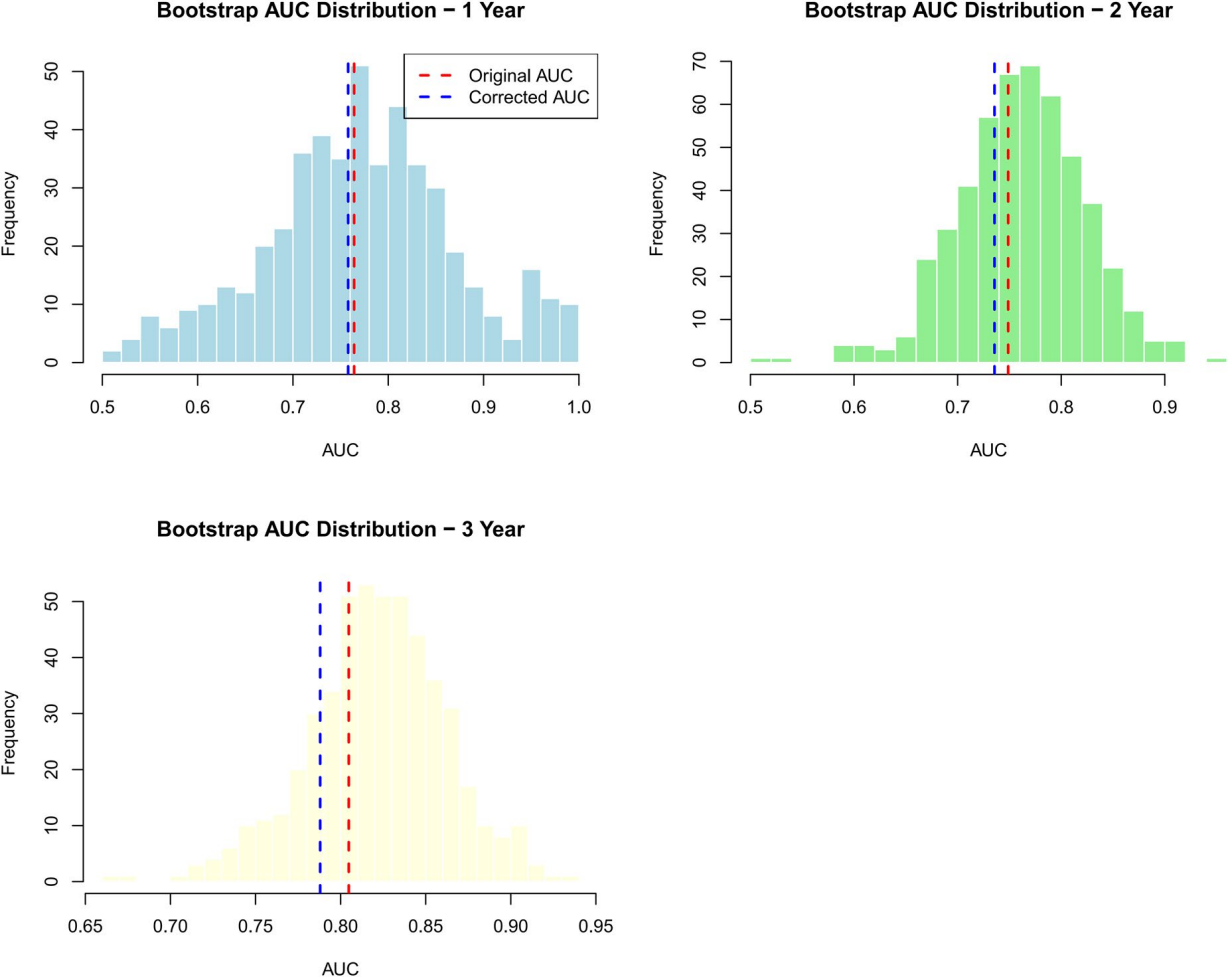
Internal validation of the nomogram using bootstrap resampling. Distribution of AUCs from 1,000 bootstrap replicates for one-year, two-year, and three-year mortality prediction. The dashed vertical lines represent the optimism-corrected AUC values.

To comprehensively evaluate the robustness and clinical utility of the predictive model, we performed a series of internal validation and model comparison analyses. First, internal validation was conducted using the bootstrap resampling method (1,000 repetitions). The results showed that the original areas under the curve (AUCs) of the model for predicting one-year, two-year, and three-year mortality were 0.764, 0.749, and 0.805, respectively. A sensitivity analysis, excluding two patients with early mortality (<6 months), confirmed the model’s robustness for medium-to-long-term prediction, showing sustained AUCs of 0.716 and 0.793 for two- and three-year mortality, respectively. After bootstrap optimism-correction, the calibrated AUCs were 0.758, 0.736, and 0.788, respectively, indicating good internal validity and a low risk of overfitting. The distribution of AUCs from bootstrap replicates is shown in Figure 5, demonstrating the stability of the model’s performance across resampled datasets. Subsequently, to quantify the incremental predictive value of our model over traditional clinical models, we constructed a baseline model incorporating only conventional clinical variables (diabetes mellitus and DBP_2h_). The full model integrating HRV/SKNA parameters demonstrated significantly improved risk discrimination over the baseline model, with an integrated discrimination improvement (IDI) index of 0.066 (*p* < .001). A positive, though statistically non-significant, net reclassification improvement (NRI) of 0.116 (*p* = .171) was also observed, suggesting a consistent trend toward improved risk reclassification. Furthermore, we developed a simplified predictive model containing only three core variables: diabetes mellitus, DBP_2h_, and rMSSD_240_. The significant incremental value of the full model was further confirmed by an IDI of 0.096 (*p* < .001), despite a non-significant NRI of 0.116 (*p* = .171). Collectively, these results indicate that the inclusion of dynamic autonomic parameters (ΔNNmean_30_, ΔApEn_30_, and ΔaSKNA_240_) provides additional information for mortality prediction beyond core clinical and static HRV indicators.

### Competing risk model results

**Figure 6.**
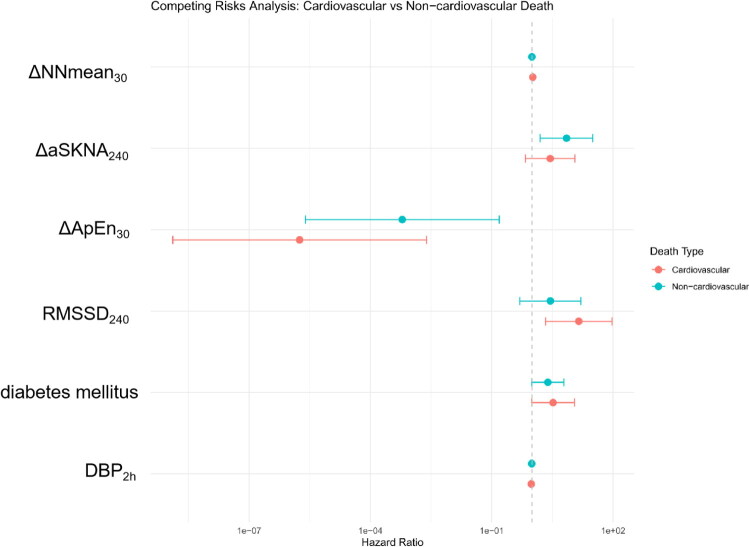
Forest plot for cause-specific mortality by univariate competing risk analysis. The forest plot displays the hazard ratios (HRs) and their confidence intervals for each predictor in cardiovascular mortality and non-cardiovascular mortality. All estimates were derived from univariate competing risk models.

Given that hemodialysis patients face competing mortality risks, we employed a competing risks analysis to differentiate the model’s ability to predict cardiovascular versus non-cardiovascular death. The nomogram demonstrated superior predictive accuracy for cardiovascular mortality, with a C-index of 0.881 and a significant association (Gray’s test *p* = .0005). In contrast, its performance for non-cardiovascular mortality was moderate, with a C-index of 0.695 and a significant but weaker association (Gray’s test *p* = .005). This differential predictive performance, as illustrated in the accompanying forest plot (Figure 6), indicates that the model is particularly effective in identifying high-risk patients susceptible to cardiovascular death, a pathway closely associated with autonomic nervous system dysfunction.

### Subgroup analysis and interaction tests

To assess the generalizability and consistency of the predictive model across different patient populations, we conducted subgroup analyses based on age and sex. First, we evaluated the model’s performance across age strata, dichotomized at the median age of 59 years. The model demonstrated excellent discriminative ability in the ≤59 years subgroup (*n* = 100, number of events = 11), with a C-index of 0.850. In the >59 years subgroup (*n* = 98, number of events = 24), the model maintained good predictive performance, with a C-index of 0.759. Interaction tests indicated that the associations between most of the six predictors and mortality risk were consistent across age subgroups (all *P* for interaction > 0.05). However, significant interactions with age were observed for DBP_2h_ and ΔApEn_30_ (*P* for interaction = 0.022 and 0.026, respectively), suggesting that the effects of these two variables on mortality risk may vary by age. Second, we analyzed the model’s performance by sex. The model exhibited stable and good predictive ability in both male (*n* = 104, number of events = 17; C-index = 0.787) and female (*n* = 94, number of events = 18; C-index = 0.740) patients. Further interaction tests confirmed that the associations between all six predictor variables and mortality risk were not statistically different between males and females (all *P* for interaction > 0.05). Complete results of the subgroup analyses are provided in Supplementary Tables 5 and 6. In summary, the predictive model developed in this study demonstrated consistent and robust performance across different age and sex subgroups.

## Discussion

The main findings of this study are as follows: 1, non-survivors demonstrated significantly lower SDNN, SDNN_240_, TTLPWR, TTLPWR_240_, VLF, VLF_240_, LF, and LF_240_ compared to the survivors; 2, the LASSO-Cox regression model identified that 6 variables (diabetes mellitus, DBP_2h_, RMSSD_240_, ΔNnmean_30_, ΔApEn_30,_ and ΔaSKNA_240_) were incorporated into the nomogram. This model demonstrated clinically relevant performance, achieving AUCs of 0.764, 0.749, and 0.805 for one-, two-, and three-year mortality prediction, with significant risk stratification (Kaplan–Meier log-rank *p* < .0001) and robust calibration.

Consistent with previous studies linking reduced HRV to poor prognosis in HD patients [11–18], we observed significantly lower SDNN, TTLPWR, VLF, and LF at multiple timepoints in the mortality subgroup. Importantly, we extended these findings by demonstrating the predictive superiority of dynamic ANS parameters over static measurements RMSSD_240_ and ΔApEn_30_ were independent predictors of mortality, suggesting that dialysis-induced autonomic instability – particularly maladaptive sympathetic activation – may contribute to long-term risk. Similarly, ΔaSKNA_240_, reflecting the acute sympathetic response to dialysis stress, further underscores the central role of ANS dysregulation. Together with prior research, this work confirms that integrating novel HRV parameters into prognostic assessment holds substantial potential across diverse populations [19]. This perspective is further supported by the findings of Günthner, Bishop, whose work similarly indicates that dynamic physiological markers can provide unique prognostic information that static measures may fail to capture [20,21]. The combination of these key dynamic parameters – RMSSD_240_, ΔApEn_30_, and ΔaSKNA_240_ – reflect dialysis-induced autonomic instability. We propose to define this composite as the ‘Autonomic Reactivity Mortality Index (ARMI)’. The ARMI may assist pre-dialysis triage and guide real-time adjustment of ultrafiltration targets to mitigate procedural stress. Elevated ARMI score could trigger adaptive dialysis algorithms, such as reducing ultrafiltration rates or initiating temperature-controlled dialysis, to preemptively mitigate risk.

The dynamic changes in HRV and SKNA parameters observed during dialysis likely reflect physiological stress responses induced by the dialysis procedure itself. Hemodialysis is frequently accompanied by rapid blood volume fluctuations and acute electrolyte shifts, which can trigger transient autonomic nervous system imbalance [14,22]. This autonomic instability may exacerbate cardiovascular strain and increase the risk of malignant arrhythmias, ultimately contributing to elevated long-term mortality [22] (Figure 7). Therefore, dynamic monitoring of HRV/SKNA parameters provides a real-time autonomic nervous system responses during dialysis and offers a physiological basis for early identification of high-risk patients and implementation of targeted interventions.

ApEn quantifies the irregularity of physiological time-series signals, where reduced ApEn values (indicating higher signal regularity) are associated with compromised ANS function or disease progression. Notably, in HD patients with concurrent coronary artery disease, an ApEn threshold <0.77 has been established as an independent predictor of cardiac mortality (RR = 4.0, *p* < .01) [23]. Extending these findings, our study demonstrated that △ApEn30 significantly predicts all-cause mortality in stage 5 CKD patients receiving HD (HR = 4.49, *p* = .013). Mechanistically, this association may reflect maladaptive sympathetic overactivation during initial dialysis phases, where transient increases in HRV complexity (elevated ApEn) signify failed compensatory mechanisms rather than physiological adaptation.

The application of LASSO regression played a pivotal role in refining the multidimensional dataset into a concise yet prognostically robust model. By applying L1-norm regularization to 91 dynamic HRV/SKNA parameters and clinical variables for feature selection, this method effectively mitigated overfitting and multicollinearity while systematically compressing the coefficients of non-predictive variables to zero, thereby accurately retaining six predictors with significant prognostic value: (diabetes mellitus, DBP2h, RMSSD240, ΔNnmean30, ΔApEn30 and ΔaSKNA240) [24]. The nomogram constructed based on these predictors demonstrated excellent discriminatory ability for one to three years mortality prediction (AUC: 0.764–0.805) and remained robust upon internal bootstrap validation (optimism-corrected AUC: 0.736–0.788). The model effectively stratified patients into distinct risk categories (Kaplan–Meier, *p* < .0001), showed strong calibration and clinical utility in calibration curve and decision curve analyses, and exhibited particularly high predictive performance for cardiovascular mortality in the competing risk model (C-index = 0.881). Consistent performance across age and sex subgroups further confirmed the model’s generalizability. By capturing dynamic autonomic markers reflective of dialysis-induced stress-such as sympathetic activation and heart rate variability complexity shifts – this LASSO-optimized nomogram provides a practical and interpretable clinical tool for personalized risk assessment in hemodialysis patients.

Our study presented several notable strengths: First, we pioneered the integration of autonomic function indices with multi-timepoint dynamic monitoring to comprehensively capture ANS responses to dialysis stress. This approach addresses a critical limitation of traditional risk stratification tools such as the Charlson Comorbidity Index [25], which rely solely on static clinical variables and fail to incorporate dynamic autonomic parameters like HRV and SKNA. Second, to address the challenge posed by numerous HRV and aSKNA parameters, we employed LASSO regression to identify key variables, thereby optimizing model simplicity and predictive performance. Third, we developed a clinically practical nomogram that combines biological and clinical variables into an intuitive visual tool for assessing all-cause mortality risk in CKD patients undergoing HD. Furthermore, the autonomic reactivity profiles established in this study provide a foundation for dual objectives: advancing precision risk stratification and supplying a rationale for developing neuromodulation strategies for autonomic dysregulation.

The findings of this study provide a critical foundation for integrating emerging wearable technologies into risk management for hemodialysis patients. As noted by Kooman et al. wearable devices hold great potential for monitoring actionable parameters in dialysis patients, yet their clinical utility depends on validated intelligent models capable of interpreting complex physiological signals [26]. The predictive model developed in this study, along with the key dynamic parameters identified directly addresses this need. Particularly noteworthy is the significant translational potential of the key autonomic predictors in our model: these parameters can not only be continuously captured using wearable ECG patches or future neuECG SKNA-enabled devices but can also be readily integrated into consumer-grade wearable devices (e.g., smartwatches, ECG patches). This characteristic enables the establishment of a continuous dynamic autonomic monitoring system, allowing for comprehensive risk assessment that extends from the dialysis center to home hemodialysis settings and even daily living environments, thereby providing robust support for intradialytic telemonitoring and early intervention. Moreover, our findings provide the physiological foundation for ‘Digital Twin Dialysis’ – virtual models that simulate patient-specific hemodynamic responses – enabling a shift from reactive to proactive, physiology-guided personalized management.

This study also has several limitations that should be acknowledged. First, although we enrolled 198 patients, the sample size remains relatively small, potentially limiting the generalizability of our findings. Second, this study stratified mortality endpoints into cardiovascular and non-cardiovascular causes. Preliminary analyses suggested distinct risk patterns, with the model showing promising predictive value, particularly for cardiac mortality. However, the constrained number of death events limited definitive conclusions. To address these limitations and build upon our findings, our future work will pursue three interconnected directions: (1) validating and improving the model’s performance for cardiovascular mortality in larger, multi-center cohorts, (2) employing privacy-preserving federated learning pipelines across dialysis centers to enable robust external validation without data sharing, and (3) exploring advanced AI techniques, such as transformer-based sequence modeling, to identify complex autonomic deterioration patterns beyond conventional regression approaches.

## Methods

### Study population

In this prospective cohort study, 198 patients undergoing maintenance HD were enrolled from Nanjing Pukou People’s Hospital (Center 1) and the First Affiliated Hospital with Nanjing Medical University (Center 2) in China from August 2021 to August 2023. The exclusion criteria were as follows (1): less than 18 years old (2); had received HD for less than three months from initiation (3); presence of fever, infection, or pregnancy (4); fasting blood glucose over 11.1 mmol/L (5); severe hepatic or pulmonary diseases, malignant tumors, or severe mental disorders (6); episodes of acute myocardial infarction, stroke, or a major surgical procedure within the past three months (7). refused the ECG and SKNA recordings. The study protocol was approved by the local Institutional Review Board, and all the participants have provided written consent. Ultimately, 198 patients were included (98 from the First Affiliated Hospital with Nanjing Medical University and 100 from Nanjing Pukou People’s Hospital). The primary outcome in our study was all-cause mortality.

### Data acquirement and processing

HD was performed under standardized conditions for all participants. The dialysis machines consisted of Baxter Gambro AK96, AK98, and Fresenius 4008S machines equipped with SSM160 and SSM180 high-flux dialyzers. A uniform blood flow rate of 250 mL/min was maintained for all procedures, with identical dialysis fluid composition administered to all patients.

Fasting venous blood samples were collected pre-dialysis in the morning and analyzed for hemoglobin, albumin, alkaline phosphatase, total cholesterol, triglycerides, serum potassium, calcium, phosphorus, intact parathyroid hormone (iPTH), and C-reactive protein (CRP). Blood pressure was measured hourly after HD initiation.

SKNA and HRV were derived from single-lead high-frequency ECG, which was recorded continuously in all patients using homemade devices published previously [9]. Signals were acquired *via* 3 M Red Dot Monitoring Electrodes (#2570) positioned at the left subclavian, right subclavian, and right lower abdominal regions, with patients maintaining a supine position to minimize motion artifacts. Raw data were bandpass filtered (500–1,000 Hz) for calculation of aSKNA as published before [10]. HRV parameters were derived using the PhysioNet Cardiovascular Signal Toolbox by Vest et al. [27] including mean heart rate (MHR), mean of all normal-to-normal intervals during 24 h (NNmean), standard deviation of normal-to-normal intervals (SDNN), root mean square of successive differences (RMSSD), total power (TTLPWR), high-frequency power (HF), low-frequency power (LF), very low-frequency power (VLF), ultra low-frequency power (ULF), the ratio of low to high-frequency power (LF/HF), acceleration capacity (AC), deceleration capacity (DC), sample entropy (SampEn), and approximate entropy (ApEn).

Five-minute (5-min) HRV and aSKNA measurements were obtained at the start of dialysis (0 min), at the 30 min, and at 240 min of dialysis. Change calculations were performed for all parameters. For example, aSKNA represented the baseline value (0 min), while values at 30 and 240 min were denoted as aSKNA_30_ and aSKNA_240_, respectively. The change, referred to as ΔaSKNA_30_, was calculated as: (aSKNA_30_-aSKNA_0_)/aSKNA_0_. This calculation method was consistently applied to all other parameters.

### Follow-up

Patients were followed until death or 30 December 2024. Survival time was recorded from the day of the 5-min ECG recording until death or the end of the follow-up period on 30 December 2024. All baseline data in the present analysis referred to the time when the 5-min electrocardiogram recording was done.

### Statistical analyses

Categorical variables are presented as frequencies (percentages). Normally distributed continuous variables are expressed as mean ± standard deviation, while non-normally distributed variables are reported as medians with interquartile ranges (IQRs). Group comparisons were performed using chi-square tests for categorical variables and Student’s t-tests or Mann–Whitney U tests for continuous variables, based on data distribution.

Analyses on prognostic factors were performed using multivariate Cox analyses and LASSO regression. LASSO-selected variables were incorporated into a multivariate logistic regression model. The final model was constructed through stepwise regression using the minimum Akaike information criterion (AIC). Model performance was evaluated by (1): generating receiver operating characteristic (ROC) curves and calculating AUC (2); assessing calibration by comparing predicted versus observed mortality (3); generating survival curves using the Kaplan-Meier method with log-rank tests for group comparisons (4); performing decision curve analysis (DCA) to determine clinical utility (5); internal validation performed using bootstrap resampling (1,000 repetitions) to estimate optimism-corrected AUCs (6); competing risk analysis conducted to differentiate cardiovascular from non-cardiovascular mortality using Gray’s test; and (7) subgroup analyses performed by age and sex, with interaction tests to assess consistency of predictor effects. A nomogram was subsequently developed based on the final model.

All statistical analyses were performed using R software (version 4.2.1). A two-sided *p* value less than .05 was considered statistically significant.

### Reporting guidelines

This study was conducted and reported in accordance with the Transparent Reporting of a Multivariable Prediction Model for Individual Prognosis or Diagnosis (TRIPOD) statement. The TRIPOD checklist is provided in the Supplementary Materials [28].

## Supplementary Material

TripodCheclist.docx

Supplementary_tables.docx

## Data Availability

The data underlying this article will be shared on reasonable request to the corresponding author.
