# Supplementary material for: Mortality prediction in hemodialysis patients using heart rate variability and skin sympathetic nerve activity
Source: Ren Fail. 2026 Jan 20;47(1):2596442. doi: 10.1080/0886022X.2025.2596442 (PMC12821348; doi:10.1080/0886022X.2025.2596442)
Supplement: TripodCheclist.docx [file IRNF_A_2596442_SM2013.docx]

| **Section/Topic** | **Item** | **Checklist Item** | **Page** |
| --- | --- | --- | --- |
| **Title and abstract** | | | |
| Title | 1 | The title identifies the study as the development and internal validation of a prediction model, specifying the target population (hemodialysis patients) and the outcome to be predicted (all-cause mortality). | 1 |
| Abstract | 2 | Summarizes the study objectives, design, setting, sample size, predictors, outcome, statistical methods, results, and conclusions. | 1 |
| **Introduction** | | | |
| Background and objectives | 3a | Describes the medical context of high mortality in hemodialysis patients, notes the limitations of existing tools, and cites relevant guidelines and literature. | 2 |
|  | 3b | Specifies the study objectives, including that the study involved both developing and internally validating a nomogram model integrating dynamic HRV/SKNA indicators to predict all-cause mortality in hemodialysis patients. | 2 |
| **Methods** | | | |
| Source of data | 4a | Describes the data source as a prospective cohort study, with data from two centers in China. | 8 |
|  | 4b | Specifies the data collection period (August 2021 to August 2023) and the end of follow-up (December 30, 2024). | 8 |
| Participants | 5a | Describes the study setting as two medical centers and specifies the geographical location (Nanjing, China). | 8 |
|  | 5b | Details the inclusion and exclusion criteria for study participants. | 8 |
|  | 5c | Describes the specific details of the standardized hemodialysis treatment received by all participants (machines, blood flow rate, dialysate, etc.). | 8 |
| Outcome | 6a | Clearly defines the primary outcome as all-cause mortality and describes the follow-up method (until death or December 30, 2024). | 8 |
|  | 6b | Not blinded. |  |
| Predictors | 7a | Clearly defines all predictors, including clinical variables, vital signs, and HRV and SKNA parameters (including baseline values and dynamic changes), and describes the measurement method and time points (0, 30, 240 minutes into dialysis). | 8 |
|  | 7b | Blinding of predictor assessors not explicitly mentioned. |  |
| Sample size | 8 | Sample size was not pre-determined. |  |
| Missing data | 9 | No missing data. |  |
| Statistical analysis methods | 10a | Describes how predictors were handled: presentation of continuous and categorical variables, and statistical methods for group comparisons. | 9 |
|  | 10b | Details the model type (logistic regression, Cox regression), modeling process (variable selection using LASSO), and internal validation method (bootstrap resampling). | 9 |
|  | 10d | Details all methods used to assess model performance (or compare models): AUC, calibration curve, Kaplan-Meier analysis, Decision Curve Analysis (DCA), internal validation via bootstrap, competing risk analysis, subgroup analysis. | 9 |
| Risk groups | 11 | Describes risk stratification (high/low risk groups) based on the total nomogram score, used in Kaplan-Meier analysis. | 4 |
| **Results** | | | |
| Participants | 13a | Describes the participant flow, including a flowchart (Figure 1) showing the number of patients finally included from the two centers. | 3 |
|  | 13b | Describes the baseline characteristics of the participants (Table 1), including demographics, clinical features, and predictors, and compares differences between the deceased and survival groups. | 3 |
| Model development | 14a | Specifies the number of participants (n=198) and outcome events (deaths=35) in each analysis. | 3 |
|  | 14b | Reports the unadjusted association between each candidate predictor and the outcome via univariable analysis (Table 1, Table 2). | 3,4 |
| Model specification | 15a | Presents the full predictive model: the nomogram (Figure 4A) visualizes the coefficient weights of all predictor variables. | 4 |
|  | 15b | Explains how to use the nomogram to calculate the 1-year, 2-year, and 3-year mortality risk for an individual patient. | 4 |
| Model performance | 16 | Reports model performance measures in the development set and internal bootstrap validation: AUC values with confidence intervals, calibration curves, C-index (for competing risk model), IDI/NRI (for model comparison). | 4,5 |
| **Discussion** | | | |
| Limitations | 18 | Discusses study limitations, including the relatively small sample size, limited number of events, and lack of external validation. | 7 |
| Interpretation | 19b | Provides an overall interpretation of the results, considering the objectives, limitations, results from other similar studies (HRV and prognosis), and other relevant evidence. | 6,7 |
| Implications | 20 | Discusses the potential clinical application of the model (early identification of high-risk patients, guiding personalized intervention) and implications for future research (integration with wearable devices, validation in larger cohorts). | 7 |
| **Other information** | | | |
| Supplementary information | 21 | States that data are available upon reasonable request and mentions the provision of supplementary tables. | 9 |
| Funding | 22 | Provides sources of funding and the role of the funders (none) and states there are no conflicts of interest. | 9 |

We recommend using the TRIPOD Checklist in conjunction with the TRIPOD Explanation and Elaboration document.
