# Supplementary material for: Mortality prediction in hemodialysis patients using heart rate variability and skin sympathetic nerve activity
Source: Ren Fail. 2026 Jan 20;47(1):2596442. doi: 10.1080/0886022X.2025.2596442 (PMC12821348; doi:10.1080/0886022X.2025.2596442)
Supplement: Supplementary_tables.docx [file IRNF_A_2596442_SM2012.docx]

**Supplementary 1. Baseline HRV indexes in Survivors and Non-survivors of CKD5 Patients.**

| **Heart rate variability** | **Overall (n=198)** | **Survivors**  **(n=163)** | **Non-survivors (n=35)** | ***P*-value** |
| --- | --- | --- | --- | --- |
|  |  |  |  |  |
|  |  |  |  |  |
| **MHR (bpm)** | 74.63±11.11 | 74.85±11.13 | 73.57±11.09 | 0.538 |
| **Time domain measures** |  |  |  |  |
| SDNN (ms) | 26.41 (20.17, 36.68) | 28.26 (20.71, 38.38) | 21.98 (18.57, 30.36) | **0.041** |
| rMSSD (ms) | 21.77 (13.76, 34.20) | 21.75 (13.78, 34.41) | 22.01 (13.75, 31.74) | 0.885 |
| NNmean (ms) | 808.93 (734.67, 906.92) | 800.68 (736.19, 916.75) | 843.52 (714.09, 878.19) | 0.745 |
| **Frequency domain measures** |  |  |  |  |
| TTLPWR | 726.72 (420.81, 1388.05) | 812.71 (460.15, 1455.05) | 495.10 (345.38, 945.83) | **0.012** |
| ULF | 48.26 (18.52, 115.31) | 48.38 (17.40, 114.69) | 40.85 (20.83, 129.09) | 0.833 |
| VLF | 321.73 (138.97, 558.53) | 344.16 (171.30, 574.61) | 151.09 (97.87, 312.99) | **<0.001** |
| LF | 127.02 (59.36, 281.92) | 146.18 (67.82, 307.16) | 61.79 (29.84, 136.09) | **<0.001** |
| HF | 130.38 (51.19, 303.15) | 122.45 (52.36, 305.01) | 139.61 (48.95, 271.96) | 0.957 |
| LF/HF | 0.92 (0.48, 1.77) | 1.07 (0.57, 1.92) | 0.57 (0.26, 0.91) | **<0.001** |
| AC | -3.79 (-5.52, -2.42) | -4.00 (-5.81, -2.61) | -2.93 (-4.71, -2.10) | 0.077 |
| DC | 3.58 (2.47, 5.40) | 3.78 (2.68, 5.64) | 2.94 (2.08, 3.99) | 0.068 |
| **Non-linear** |  |  |  |  |
| SampEn | 1.47±0.42 | 1.47±0.41 | 1.48±0.50 | 0.927 |
| ApEn | 1.07 (0.96, 1.15) | 1.08 (0.97, 1.15) | 1.02 (0.87, 1.14) | 0.149 |
| **aSKNA** | 1.03 (0.86, 1.25) | 1.05 (0.88, 1.25) | 0.96 (0.81, 1.21) | 0.272 |

**Abbreviations:** HRV, heart rate variability; MHR, mean heart rate; SDNN, standard deviation of normal-to-normal intervals; rMSSD, root mean square of successive differences; NNmean, mean of all normal-to-normal intervals during 24 hours; TTLPWR, total power; ULF, ultra low-frequency power; VLF, very low frequency; LF, low frequency; HF, high frequency; AC, acceleration capacity; DC, deceleration capacity; SampEn, sample entropy; ApEn, approximate entropy; aSKNA, average amplitude of skin sympathetic nerve activity. Data are presented as mean ± SD, numbers and percentages, as appropriate.

**Supplementary 2. After 30min dialysis of HRV Indexes in Survivors and Non-survivors of CKD5 Patients.**

| **Heart rate variability** | **Overall (n=198)** | **Survivors (n=163)** | **Non-survivors (n=35)** | ***P*-value** |
| --- | --- | --- | --- | --- |
|  |  |  |  |  |
|  |  |  |  |  |
| **MHR (bpm)** | 74.20±12.07 | 74.04±12.06 | 74.97±12.25 | 0.678 |
| **Time domain measures** |  |  |  |  |
| SDNN (ms) | 22.64 (16.50, 32.94) | 23.48 (16.29, 34.12) | 21.40 (17.12, 26.93) | 0.445 |
| rMSSD (ms) | 16.12 (10.16, 26.19) | 15.59 (9.84, 26.55) | 17.07 (14.14, 25.17) | 0.455 |
| NNmean (ms) | 817.73 (745.51, 918.27) | 817.27 (748.42, 923.64) | 819.70 (744.36, 896.20) | 0.765 |
| **Frequency domain measures** |  |  |  |  |
| TTLPWR | 600.31 (287.38, 1239.06) | 618.54 (285.92, 1302.38) | 473.03 (293.36, 829.11) | 0.226 |
| ULF | 28.59 (8.02, 79.44) | 29.98 (9.06, 77.41) | 21.55 (5.16, 71.45) | 0.292 |
| VLF | 264.83 (131.30, 465.86) | 274.47 (134.89, 477.24) | 239.88 (114.14, 414.14) | 0.341 |
| LF | 103.76 (37.41, 257.01) | 124.14 (37.73, 278.27) | 78.62 (39.32, 143.76) | 0.112 |
| HF | 91.61 (37.75, 218.70) | 91.23 (34.04, 227.70) | 91.99 (45.71, 139.86) | 0.871 |
| LF/HF | 1.06 (0.52, 2.22) | 1.10 (0.54, 2.27) | 0.98 (0.48, 1.92) | 0.307 |
| AC | -4.04 (-6.48, -2.44) | -4.24 (-6.72, -2.44) | -3.46 (-5.09, -2.49) | 0.306 |
| DC | 4.19 (2.61, 6.27) | 4.37 (2.72, 6.40) | 3.26 (2.39, 4.75) | 0.078 |
| **Non-linear** |  |  |  |  |
| SampEn | 1.57 (1.25, 1.95) | 1.56 (1.27, 1.92) | 1.58 (1.13, 2.07) | 0.755 |
| ApEn | 1.08 (0.96, 1.21) | 1.08 (0.98, 1.21) | 1.06 (0.92, 1.22) | 0.567 |
| **aSKNA** | 1.03 (0.84, 1.34) | 1.05 (0.84, 1.36) | 0.99 (0.90, 1.21) | 0.418 |

**Abbreviations:** HRV, heart rate variability; MHR, mean heart rate; SDNN, standard deviation of normal-to-normal intervals; rMSSD, root mean square of successive differences; NNmean, mean of all normal-to-normal intervals during 24 hours; TTLPWR, total power; ULF, ultra low-frequency power; VLF, very low frequency; LF, low frequency; HF, high frequency; AC, acceleration capacity; DC, deceleration capacity; SampEn, sample entropy; ApEn, approximate entropy; aSKNA, average amplitude of skin sympathetic nerve activity. Data are presented as mean ± SD, numbers and percentages, as appropriate.

**Supplementary 3. Delta 30min_baseline HRV Indexes in Survivors and Non-survivors of CKD5 Patients.**

| **Heart rate variability** | **Overall (n=198)** | **Survivors (n=163)** | **Non-survivors (n=35)** | ***P*-value** |
| --- | --- | --- | --- | --- |
|  |  |  |  |  |
|  |  |  |  |  |
| **MHR (bpm)** | -0.01 (-0.04, 0.02) | -0.01 (-0.05, 0.01) | 0.01 (-0.01, 0.05) | **<0.001** |
| **Time domain measures** |  |  |  |  |
| SDNN (ms) | -0.10 (-0.29, 0.08) | -0.10 (-0.26, 0.09) | -0.13 (-0.37, 0.03) | 0.283 |
| rMSSD (ms) | -0.12 (-0.45, 0.15) | -0.10 (-0.44, 0.16) | -0.27 (-0.45, 0.03) | 0.091 |
| NNmean (ms) | 0.01 (-0.02, 0.04) | 0.01 (-0.02, 0.04) | 0.01 (-0.03, 0.05) | 0.845 |
| **Frequency domain measures** |  |  |  |  |
| TTLPWR | -0.22 (-0.64, 0.58) | -0.26 (-0.65, 0.56) | 0.06 (-0.59, 0.74) | 0.339 |
| ULF | -0.39 (-0.84, 1.67) | -0.39 (-0.83, 1.65) | -0.54 (-0.88, 1.17) | 0.451 |
| VLF | -0.15 (-0.40, 0.29) | -0.15 (-0.42, 0.28) | -0.18 (-0.39, 0.29) | 0.915 |
| LF | -0.08 (-0.49, 0.42) | -0.09 (-0.47, 0.43) | -0.07 (-0.57, 0.30) | 0.409 |
| HF | -0.10 (-0.61, 0.35) | -0.06 (-0.58, 0.54) | -0.35 (-0.68, 0.10) | **0.044** |
| LF/HF | -0.03 (-0.33, 0.82) | -0.08 (-0.34, 0.78) | 0.16 (-0.29, 1.17) | 0.355 |
| AC | 0.04 (-0.16, 0.36) | 0.04 (-0.15, 0.33) | 0.02 (-0.19, 0.38) | 0.843 |
| DC | 0.13 (-0.14, 0.39) | 0.13 (-0.14, 0.38) | 0.04 (-0.14, 0.41) | 0.640 |
| **Non-linear** |  |  |  |  |
| SampEn | 0.06 (-0.07, 0.28) | 0.05 (-0.08, 0.27) | 0.08 (-0.03, 0.56) | 0.361 |
| ApEn | 0.01 (-0.05, 0.11) | 0.01 (-0.05, 0.10) | 0.03 (-0.06, 0.15) | 0.507 |
| **aSKNA** | -0.01 (-0.11, 0.17) | -0.01 (-0.11, 0.18) | -0.01 (-0.12, 0.16) | 0.987 |

**Abbreviations:** HRV, heart rate variability; MHR, mean heart rate; SDNN, standard deviation of normal-to-normal intervals; rMSSD, root mean square of successive differences; NNmean, mean of all normal-to-normal intervals during 24 hours; TTLPWR, total power; ULF, ultra low-frequency power; VLF, very low frequency; LF, low frequency; HF, high frequency; AC, acceleration capacity; DC, deceleration capacity; SampEn, sample entropy; ApEn, approximate entropy; aSKNA, average amplitude of skin sympathetic nerve activity. Data are presented as mean ± SD, numbers and percentages, as appropriate.

**Supplementary 4. After 240min dialysis of HRV Indexes in Survivors and Non-survivors of CKD5 Patients.**

| **Heart rate variability** | **Overall (n=198)** | **Survivors (n=163)** | **Non-survivors (n=35)** | ***P*-value** |
| --- | --- | --- | --- | --- |
|  |  |  |  |  |
|  |  |  |  |  |
| **MHR (bpm)** | 80.51±13.58 | 80.56±13.87 | 80.28±12.32 | 0.913 |
| **Time domain measures** |  |  |  |  |
| SDNN (ms) | 26.07 (18.62, 36.70) | 27.73 (19.24, 37.44) | 20.49 (15.82, 31.21) | **0.021** |
| rMSSD (ms) | 19.06 (11.74, 28.80) | 18.23 (10.97, 27.14) | 23.60 (15.90, 33.99) | 0.052 |
| NNmean (ms) | 765.71±126.71 | 766.38±128.14 | 762.66±119.94 | 0.874 |
| **Frequency domain measures** |  |  |  |  |
| TTLPWR | 744.71 (356.38, 1399.98) | 889.39 (444.52, 1577.78) | 352.52 (233.64, 852.02) | **0.001** |
| ULF | 115.54±263.90 | 119.95±274.06 | 95.45±210.32 | 0.616 |
| VLF | 358.61 (161.61, 731.43) | 404.20 (199.48, 798.67) | 160.57 (90.64, 300.03) | **<0.001** |
| LF | 141.55 (58.46, 311.85) | 159.58 (74.42, 351.35) | 47.95 (28.32, 160.55) | **<0.001** |
| HF | 107.33 (43.00, 286.68) | 106.77 (42.22, 287.41) | 107.90 (55.37, 261.14) | 0.607 |
| LF/HF | 1.13 (0.58, 2.84) | 1.38 (0.68, 3.15) | 0.54 (0.29, 0.96) | **<0.001** |
| AC | -3.64 (-5.96, -2.33) | -3.83 (-6.33, -2.44) | -3.11 (-4.53, -1.90) | 0.058 |
| DC | 3.49 (2.21, 5.77) | 3.86 (2.51, 5.99) | 2.35 (1.59, 3.69) | **0.002** |
| **Non-linear** |  |  |  |  |
| SampEn | 1.42±0.42 | 1.40±0.41 | 1.51±0.44 | 0.146 |
| ApEn | 1.08 (0.94, 1.18) | 1.07 (0.93, 1.17) | 1.15 (0.99, 1.22) | 0.067 |
| **aSKNA** | 1.00 (0.86, 1.20) | 0.99 (0.86, 1.18) | 1.05 (0.88, 1.24) | 0.336 |

**Abbreviations:** HRV, heart rate variability; MHR, mean heart rate; SDNN, standard deviation of normal-to-normal intervals; rMSSD, root mean square of successive differences; NNmean, mean of all normal-to-normal intervals during 24 hours; TTLPWR, total power; ULF, ultra low-frequency power; VLF, very low frequency; LF, low frequency; HF, high frequency; AC, acceleration capacity; DC, deceleration capacity; SampEn, sample entropy; ApEn, approximate entropy; aSKNA, average amplitude of skin sympathetic nerve activity. Data are presented as mean ± SD, numbers and percentages, as appropriate.

**Supplementary 5. Delta 240min_baseline HRV Indexes in Survivors and Non-survivors of CKD5 Patients.**

| **Heart rate variability** | **Overall (n=198)** | **Survivors (n=163)** | **Non-survivors (n=35)** | ***P*-value** |
| --- | --- | --- | --- | --- |
|  |  |  |  |  |
|  |  |  |  |  |
| **MHR (bpm)** | 0.07 (-0.01, 0.15) | 0.05 (-0.01, 0.15) | 0.08 (0.02, 0.16) | 0.242 |
| **Time domain measures** |  |  |  |  |
| SDNN (ms) | -0.03 (-0.22, 0.29) | -0.01 (-0.23, 0.29) | -0.06 (-0.20, 0.30) | 0.640 |
| rMSSD (ms) | -0.14 (-0.43, 0.42) | -0.17 (-0.45, 0.37) | 0.09 (-0.29, 0.72) | 0.051 |
| NNmean (ms) | －0.06±0.12 | －0.06±0.12 | －0.06±0.08 | 0.690 |
| **Frequency domain measures** |  |  |  |  |
| TTLPWR | 0.02 (-0.40, 0.76) | 0.07 (-0.38, 0.80) | -0.21 (-0.41, 0.36) | 0.118 |
| ULF | 6.31±32.34 | 7.13±35.48 | 2.58±7.62 | 0.447 |
| VLF | 0.10 (-0.42, 1.09) | 0.15 (-0.36, 1.23) | -0.09 (-0.52, 0.45) | 0.081 |
| LF | 0.26 (-0.44, 1.18) | 0.28 (-0.43, 1.18) | 0.03 (-0.54, 1.19) | 0.580 |
| HF | -0.18 (-0.62, 0.94) | -0.20 (-0.63, 0.87) | -0.12 (-0.53, 1.27) | 0.489 |
| LF/HF | 0.32 (-0.32, 1.64) | 0.36 (-0.29, 1.67) | 0.06 (-0.52, 1.52) | 0.265 |
| AC | -0.05 (-0.39, 0.44) | -0.03 (-0.39, 0.45) | -0.08 (-0.37, 0.34) | 0.992 |
| DC | 0.02 (-0.35, 0.46) | 0.03 (-0.35, 0.51) | -0.16 (-0.42, 0.28) | 0.122 |
| **Non-linear** |  |  |  |  |
| SampEn | -0.02 (-0.23, 0.24) | -0.03 (-0.24, 0.22) | 0.02 (-0.21, 0.36) | 0.294 |
| ApEn | 0.01 (-0.11, 0.12) | 0.00 (-0.11, 0.10) | 0.10 (-0.10, 0.25) | **0.034** |
| **aSKNA** | -0.03 (-0.13, 0.07) | -0.04 (-0.14, 0.05) | 0.02 (-0.09, 0.14) | **0.028** |

**Abbreviations:** HRV, heart rate variability; MHR, mean heart rate; SDNN, standard deviation of normal-to-normal intervals; rMSSD, root mean square of successive differences; NNmean, mean of all normal-to-normal intervals during 24 hours; TTLPWR, total power; ULF, ultra low-frequency power; VLF, very low frequency; LF, low frequency; HF, high frequency; AC, acceleration capacity; DC, deceleration capacity; SampEn, sample entropy; ApEn, approximate entropy; aSKNA, average amplitude of skin sympathetic nerve activity. Data are presented as mean ± SD, numbers and percentages, as appropriate.

**Supplementary 5. Subgroup Analysis by Age: Model Discrimination and Univariate Cox Regression Results**

|  | Subgroup | HR (95% CI) | *P*-value | *P* for Interaction |
| --- | --- | --- | --- | --- |
| Diabetes mellitus | ≤59 years | 5.22 (1.59-17.10) | 0.006 | 0.092 |
|  | >59 years | 1.58 (0.70-3.57) | 0.268 |  |
| DBP_2h_ | ≤59 years | 1.03 (0.99-1.07) | 0.189 | 0.022 |
|  | >59 years | 0.96 (0.93-1.00) | 0.045 |  |
| △aSKNA_240_ | ≤59 years | 14.15 (1.71-117.23) | 0.014 | 0.28 |
|  | >59 years | 2.23 (0.14-36.53) | 0.575 |  |
| rMSSD_240_ | ≤59 years | 1.01 (0.99-1.03) | 0.2 | 0.578 |
|  | >59 years | 1.01 (0.99-1.02) | 0.445 |  |
| △NNmean_30_ | ≤59 years | 0.07 (<0.01-99.09) | 0.465 | 0.055 |
|  | >59 years | <0.01 (<0.01-0.003) | <0.001 |  |
| △ApEn_30_ | ≤59 years | 8.23 (1.68-40.42) | 0.009 | 0.026 |
|  | >59 years | 0.42 (0.04-4.01) | 0.451 |  |

**Abbreviations:** DBP, diastolic blood pressure; aSKNA, average skin sympathetic nerve activity; rMSSD, root mean square of successive differences; ApEn, approximate entropy; NNmean, mean of all normal-to-normal intervals during 24 hours.

**Supplementary 6. Subgroup Analysis by Sex: Model Discrimination and Univariate Cox Regression Results**

|  | Subgroup | HR (95% CI) | *P*-value | *P* for Interaction |
| --- | --- | --- | --- | --- |
| Diabetes mellitus | Male | 3.09 (1.19-8.03) | 0.020 | 0.608 |
|  | Female | 2.13 (0.84-5.41) | 0.111 |  |
| DBP_2h_ | Male | 1.00 (0.96-1.04) | 0.930 | 0.619 |
|  | Female | 0.99 (0.95-1.02) | 0.433 |  |
| △aSKNA_240_ | Male | 12.27 (1.52-99.10) | 0.019 | 0.321 |
|  | Female | 1.71 (0.12-25.47) | 0.698 |  |
| rMSSD_240_ | Male | 1.00 (0.98-1.03) | 0.766 | 0.657 |
|  | Female | 1.01 (1.00-1.02) | 0.149 |  |
| △NNmean_30_ | Male | 0.001 (<0.001-0.23) | 0.014 | 0.400 |
|  | Female | 0.03 (<0.001-17.81) | 0.286 |  |
| △ApEn_30_ | Male | 0.46 (0.03-6.90) | 0.577 | 0.204 |
|  | Female | 3.05 (0.80-11.58) | 0.101 |  |

**Abbreviations:** DBP, diastolic blood pressure; aSKNA, average skin sympathetic nerve activity; rMSSD, root mean square of successive differences; ApEn, approximate entropy; NNmean, mean of all normal-to-normal intervals during 24 hours.
